# Identification of CP12 as a Novel Calcium-Binding Protein in Chloroplasts

**DOI:** 10.3390/plants2030530

**Published:** 2013-08-26

**Authors:** Agostinho Gomes Rocha, Ute C. Vothknecht

**Affiliations:** 1 Department of Biology I, LMU Munich, Großhaderner Street 2-4, D-82152 Planegg-Martinsried, Germany; E-Mail: vothknecht@bio.lmu.de; 2 Center for Integrated Protein Science (Munich) at the Department of Biology of the LMU Munich, D-81377 Munich, Germany

**Keywords:** chloroplast, calcium signaling, CP12, Calvin-Benson-Bassham Cycle

## Abstract

Calcium plays an important role in the regulation of several chloroplast processes. However, very little is still understood about the calcium fluxes or calcium-binding proteins present in plastids. Indeed, classical EF-hand containing calcium-binding proteins appears to be mostly absent from plastids. In the present study we analyzed the stroma fraction of Arabidopsis chloroplasts for the presence of novel calcium-binding proteins using 2D-PAGE separation followed by calcium overlay assay. A small acidic protein was identified by mass spectrometry analyses as the chloroplast protein CP12 and the ability of CP12 to bind calcium was confirmed with recombinant proteins. CP12 plays an important role in the regulation of the Calvin-Benson-Bassham Cycle participating in the assembly of a supramolecular complex between phosphoribulokinase and glyceraldehyde 3-phosphate dehydrogenase, indicating that calcium signaling could play a role in regulating carbon fixation.

## 1. Introduction

Chloroplasts are key organelles for plant autotrophism, where many essential metabolic processes take place. In addition to photosynthesis, plastids perform functions such as fatty acid biosynthesis, nitrite and sulphate reduction and amino acid biosynthesis. To ensure proper function and regulation, chloroplast metabolism is tightly coordinated with the requirements of the surrounding cell. Accordingly, environmental and developmental signals have to be transduced into the organelle. Calcium is a ubiquitous secondary messenger of eukaryotic organisms involved in the regulation of multiple cellular processes [1,2]. In chloroplasts, calcium has been shown to be involved in the regulation of processes such as photosynthesis, CO_2_ fixation, protein transport and protein phosphorylation. As recently reviewed, about twenty proteins are so far known to be potentially involved in the chloroplast calcium network, however, only a few have been shown to bind calcium in a direct manner [3]. The photosystem II protein PsbO has been reported as a weak calcium-binding protein [4,5] and calcium was suggested to influence its proton-dependent activation [6]. Ferredoxin, the electron donor of photosystem I, is also able to bind calcium with high capacity in its reduced state [7]. The thylakoid localized calcium sensing protein (CAS) is one of the most extensively studied calcium-binding proteins in plastids. It is involved in stomatal closure [8,9,10,11] and photoacclimation [12] and was one of three thylakoid targets of calcium-dependent phosphorylation identified in a recent study [13]. The same study also showed Var1 and PsaN to be phosphorylated in a calcium-dependent manner.

Present in all eukaryotes, calmodulins (CaMs) are one of the best studied families of calcium-binding proteins. They are comprised of four EF-hand structural motifs that are able to bind calcium with high affinity [14] and bind to their targets in a calcium-dependent manner. Several chloroplast proteins have been shown to behave as CaM-binding proteins, including the photosystem I component PsaN [15], the chaperonine CPN10 [16], as well as the AAA^+^ proteins CIP111 and AFG1L1 [17,18]. However, in chloroplasts, no CaM has been so far identified. Indeed, typical EF-hand containing calcium-binding proteins seems to be very rare in chloroplasts. In the outer envelope, a member of the calcium-dependent mitochondrial carrier family SAMTL has been identified [19,20] that possesses a single EF-hand domain and was shown to specifically bind calcium *in vitro* [21]. Also, the (p)ppGpp synthase-degradase CRSH has an EF-hand domain and requires calcium for its activity [22]. The latter is the only stromal EF-hand protein identified so far.

In the present work, we investigated the presence of additional calcium-binding proteins in the stroma. To that end, proteins were separated by 2D SDS-PAGE and tested for the ability to bind radiolabeled calcium by calcium overlay assays. CP12 was identified as a potential candidate for calcium-binding and this property could be confirmed *in vitro* using recombinant protein. Together, our data suggest that CP12 represents a novel calcium-binding protein in chloroplasts.

## 2. Results and Discussion

### 2.1. Identification of Novel Chloroplast Calcium-Binding Proteins

**Figure 1:**
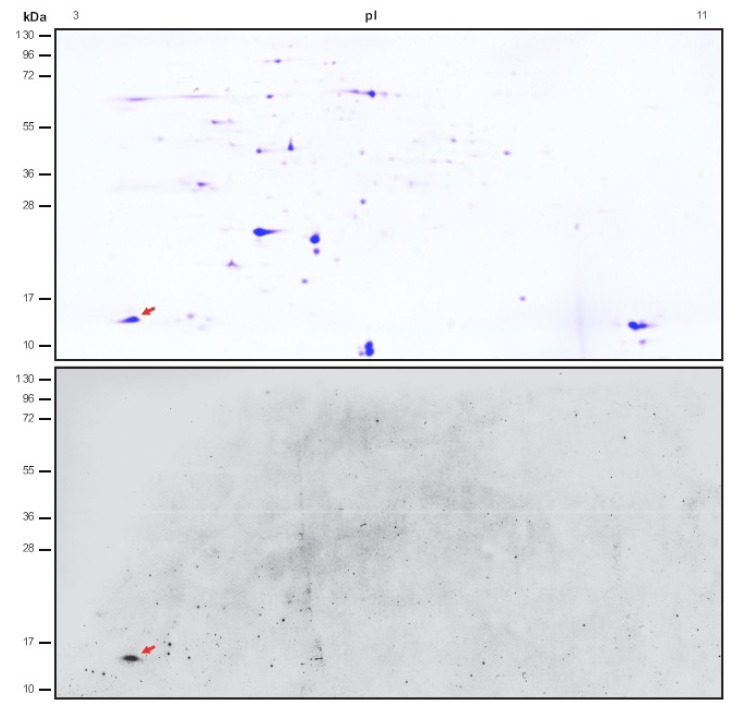
Identification of novel calcium-binding proteins in chloroplasts. Combined **s**tromal and extrinsic membrane proteins from Arabidopsis chloroplasts were separated by 2D-PAGE (IEF followed by SDS-PAGE) and assessed for calcium-binding activity using the radioactive isotope ^45^Ca. Coomassie brilliant blue stained gel (upper panel) and autoradiogram (lower panel) are shown. A potential calcium-binding protein of about 15 kDa indicated by arrows was excised and analyzed by MS/MS.

In this study we analyzed chloroplasts from Arabidopsis to identify novel calcium-binding proteins. After initial purification, chloroplasts were disrupted by treatment with hypertonic buffer and soluble and membrane proteins were separated by centrifugation. Extrinsic proteins were subsequently removed from the membrane by a high-salt wash and combined with the stromal fraction. After desalting, proteins were then separated by 2D-PAGE using IEF in the first and SDS-PAGE in the second dimension (Figure 1, upper panel). Proteins were electrophoretically transferred onto a PVDF membrane and calcium-binding ability was assessed by incubation with buffer containing radioactive isotope ^45^Ca after re-naturation of the proteins on the membrane. In order to avoid unspecific binding, the buffer contained excess of the divalent cation magnesium. Under these conditions, a single protein that clearly bound calcium was observed (Figure 1, lower panel, red arrow). This protein migrates at about 15 kDa and was focused on the acidic region of the membrane.

**Figure 2:**
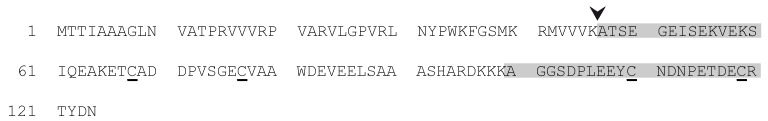
Deduced amino acid sequence of Arabidopsis CP12. Grey bars indicate peptides found by tandem mass spectroscopy that matched to this protein. An arrow indicates the potential cleavage site of the transit peptide [24]. Four conserved Cys residues of CP12 are underlined.

Coomassie brilliant blue staining of a SDS-PAGE duplicate from the same sample revealed a protein pattern different from what is normally observed in stromal fractions, notable easily by the near complete lack of RuBisCO. It appears that the desalting step removed larger proteins and protein complexes and thus enriched many of the smaller proteins. This might also explain, why only a single calcium-binding spot was observed. However, the stain showed a clear protein band in the same area where the radioactive signal was observed (Figure 1, upper panel, red arrow). The protein was excised from the gel and analyzed by MS/MS. Two peptides were found that matched the sequence of the known chloroplast protein CP12 as indicated by grey bars (Figure 2). The mature CP12 protein, after cleavage of the targeting peptide (Figure 2, indicated by arrow), has a predicted mass of 12 kDa, which fits well with the size observed on SDS-PAGE separation. In addition, the theoretical isoelectric point of 4.15 is in agreement with the observed position in the IEF separation [23].

### 2.2. CP12 Binds Calcium with High Affinity

**Figure 3:**
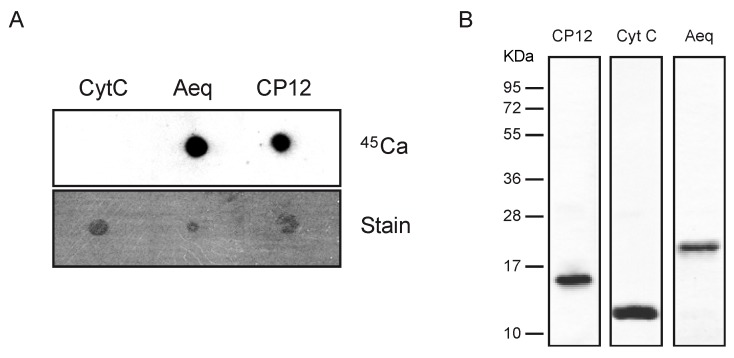
CP12 is a calcium-binding protein. (**A**) Autoradiogram of calcium-overlay assay using recombinant CP12 (upper panel). Aequorin (Aeq) and cytochrome C (CytC) were used as positive and negative controls, respectively. Four micrograms of each protein were spotted onto a PVDF membrane and post stained by coomassie brilliant blue (lower panel). (**B**) Purity of recombinant proteins used for calcium-overlay assays was assessed by SDS-PAGE and stained by coomassie brilliant blue.

To validate whether CP12 is indeed a calcium-binding protein, a calcium overlay assay was performed with recombinant protein (Figure 3). The mature protein, without targeting peptide, was cloned into the pTWIN1 vector and purified from *E. coli* under native conditions. The well-established calcium-binding protein aequorin (Aeq) and cytochrom C (CytC) were used as positive and negative controls, respectively.

**Figure S1:**
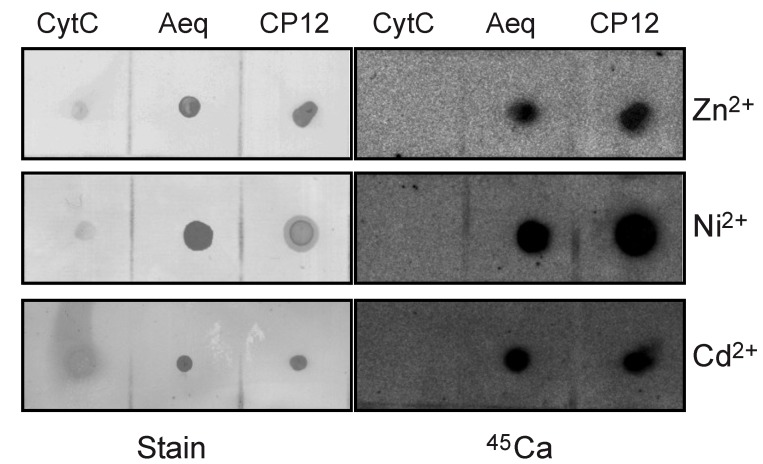
Calcium competition assays with other divalent cations. Autoradiogram of calcium-overlay assay using recombinant CP12. In addition to ^45^Ca, buffers contained either 0.1 mM zinc acetate (Zn^2+^), nickel sulphate (Ni^2+^) or cadmium acetate (Cd^2+^). Aequorin (Aeq) and cytochrome C (CytC) were used as positive and negative controls, respectively (right panel). Two µg of each protein were spotted onto a PVDF membrane and post stained by coomassie brilliant blue (left panel).

Purity of the isolated proteins was confirmed by SDS-PAGE (Figure 3B). Calcium-overlay assays with ^45^Ca were performed by direct spotting of the proteins onto a PDVF membrane and calcium-binding was analyzed by autoradiogram (Figure 3A, upper panel). Aequorin, which contains three calcium-binding EF domains shows the strongest signal even though less protein was apparently present on the membrane (Figure 3A). CytC does not display any calcium binding showing that it does not occur unspecifically with any protein. On the other hand, CP12 shows strong calcium binding albeit slightly weaker than aequorin. To further confirm specific binding of calcium to CP12, binding assay were performedin the presence of other divalent cations (Figure S1). Neither Zn^2+^, Ni^2+^ nor Cd^2+^ were able to compete for binding of ^45^Ca. All together these results indicate that CP12 is a novel chloroplast calcium-binding protein. CP12 does not contain typical calcium binding EF-hand motifs, so the exact molecular base for calcium binding needs to investigated. However, based on the protein sequence one can speculate that conserved negative charged amino acid residues that occur in abundance in the *C*-terminal part of the protein could be involved in this process.

## 3. Experimental Section

### 3.1. Plant Growth and Preparation of Chloroplast Proteins

*Arabidopsis thaliana* (cultivar Columbia Col-0) was grown on soil at 22 °C under a 16 h/8 h photoperiod at 150 μmol m^−2^·s^−1^. Chloroplasts purified from 7 weeks old Arabidopsis leafs as described in [25] were disrupted by suspension in lysis buffer (20 mM Tricine pH 7.6, 10% (v/v) glycerol, 1 mM DTT) supplemented with protease inhibitors (Complete™ EDTA free, Roche, Switzerland), phosphatase inhibitors (Phospho-Stop, Roche, Switzerland) and 5 mM EGTA. After incubation on ice for 15 min, membranes and soluble components were separated by centrifugation at 60,000 g for 10 min. To extract membrane associated proteins, the pellet was subsequently resuspended in lysis buffer containing 0.8 M NaCl and centrifuged again at 60,000 g for 10 min. Supernatants of the first and second centrifugation were combined, concentrated and desalted into lysis buffer using Vivaspin^TM^ 500 columns (GE Healthcare, Buckinghamshire, UK) and is referred to as stromal protein fraction. 

### 3.2. Proteins Separation by 2D IEF-SDS PAGE

Eighty microliters of soluble stromal protein fraction (150–250 µg) were resuspended in 400 µL rehydration buffer (7 M urea, 2 M thiourea, 4% (w/v) CHAPS, 0.7% (v/v) IPG buffer, 0.002% (w/v) bromophenol blue and 40 mM DTT) for 30 min at RT. Proteins were applied to 24 cm immobilized Dry-Strips^TM^ (pH 3-11, NL, GE Healthcare) and separated using the BioRad Protean IEF Cell Isoelectric Focusing System according to the following programme: Passive rehydratation a 20 °C for 20 h; rapid—1 h (0.15 kV); rapid—1 h (0.3 kV); rapid—1 h (0.6 kV); rapid—1 h (1 kV); linear (ramp)—1 h (10 kV); rapid—45 kV/h (10 kV); hold(1 kV). Afterwards, the strips were equilibrated consecutively in equilibration buffer containing 10 mg/mL DTT and in equilibration buffer containing 25 mg/mL iodacetamide for 20 min each. Proteins were separated in the second dimension by SDS-PAGE and electrophoretically transferred onto a PVDF membrane to be used for calcium overlay assays. For MS/MS propose, the assay was conducted in parallel but without blotting. The SDS-PAGE was stained with coomassie brilliant blue R-250 after the second dimension, and spots of interest were excised.

### 3.3. Mass Spectrometry Analysis

Protein spots were excised and prepared for mass spectrometry analysis (MS) as previously described [19]. Tryptic digests were separated on an UltiMate 3000 HPLC system (Thermo Fisher Scientific, Dionex, CA, USA). Peptides were loaded on a trapping column (PepMap C18, 5µm particle size, 300 μm i.d. × 5 mm) equilibrated with 0.1% TFA (trifluoricacetc acid) and separated on an analytical column (PepMap C18, 3 μm, 75 μm i.d. × 150 mm) applying a 60 min linear gradient from 2.5% up to 40% ACN with 0.1% formic acid. The HPLC was directly coupled to an LTQ XL linear ion trap mass spectrometer (Thermo Fisher Scientific, Dionex, CA, USA) equipped with a nanoelectrospray ionization source (Proxeon). The electrospray voltage was set to 1,500 V. The mass spectrometer was operated in the data-dependent mode: 1 full scan (*m/z*: 450–1,600) was followed by maximal 10 MS/MS scans with CID. When a neutral loss of 32.6 or 49 Da was detected, a MS3 scan was triggered. The collision energy was set at 35%, Q-value at 0.25 and the activation time at 30 ms. Fragmented ions were set onto an exclusion list for 20 s.

Raw spectra were interpreted by Mascot 2.2.04 (Matrix Science) using Mascot Daemon 2.2.2. The peptide tolerance was set to 2 Da, MS/MS tolerance was set to 0.8 Da. Spectra were searched against the Arabidopsis database from 09.09.2009 (TAIR), with tryptic specificity and 2 missed cleavages allowed. Carbamidomethyl was set as a fixed modification of cysteine, oxidation of methionine and phosphorylation of serine and threonine were set as variable modifications.

MASCOT results were loaded into Scaffold (Version 2.04, Proteome Software). Peptide identifications were accepted, if they could be established at greater than 95% probability as specified by the Peptide Prophet algorithm. To be considered as relevant, protein identifications required a minimum of two unique peptides.

### 3.4. Radioactive Calcium Overlay Assays

Assays were done as described earlier [26] with minor modifications. Briefly, stromal proteins separated by 2D-PAGE were electrophoretically transferred to a PVDF activated membrane, while recombinant proteins, including aequorin and commercially available cytochrom C (New England Biolabs GmbH, Germany), were spotted directly onto the membrane. The membrane was subsequently incubated three times for 20 min with calcium washing buffer (60 mM KCl, 5 mM MgCl_2_, 60 mM imidazole/HCl pH 6.8) followed by 10 min incubation in the same buffer containing 0.1 μM ^45^CaCl_2_ (13.90 mCi/mg; Perkin Elmer) and for competition assays, 0.1 mM “cold” zinc acetate, nickel sulphate or cadmium acetate was included in all buffers. Membranes were washed for 5 min with 50% ethanol and radioactivity signals were detected by exposure on phospho-imaging screens and analysed on a FUJI FLA-3000 (FUJIFILM). All incubation steps were performed at room temperature. 

### 3.5. Expression and Purification of Recombinant CP12

CP12 (At2g47400) lacking the *N*-terminal 47 amino acids (chloroplast targeting sequence) was cloned into pTWIN1 in frame with the *N*-terminal intein tag (forward primer: 5'-ATACCATGGCTACATCGGAAGGAGAGAT-3' and reverse primer 5'-TTAGCGGCCGCAATTATCATAAGTACGACAC-3'). The protein was expressed in *Escherichia coli* and purified under native conditions by using the IMPACT-pTWIN protein purification system (New England Biolabs GmbH), according to the manufacturer’s instructions. The intein tag was cleaved during affinity purification and after elution the buffer was exchanged to 20 mM Tricine/NaOH (7.6), 1 mM DTT and the protein was concentrated by ultrafiltration in Vivaspin 500 columns (3 kDa cutoff, GE Healthcare). Except the cleavage step, all purification steps were performed on ice or at 4 °C.

## 4. Conclusions

Calcium has been shown to influence several processes in chloroplasts, but in fact, very few calcium-binding proteins have been described so far. In this work, we investigated the presence of novel calcium-binding proteins in the stroma, where important metabolic processes, such as the carbon fixation, take place. By the approach described here, a novel calcium-binding protein could be identified. CP12 is a chloroplast localized protein widely present in photosynthetic organisms. CP12 orthologs have been identified in plants, green algae, cyanobacteria and diatoms [27,28,29,30,31,32,33]. This small chloroplast protein contains four conserved cystein residues [34,35] and serves as a linker in the reversible assembly of the supramolecular complex between phosphoribulokinase (PRK) and glyceraldehyde 3-phosphate dehydrogenase (GAPDH). This is part of a regulatory circuit in which GADH and PRK are inactivated by complex formation via CP12 [28]. From antisense studies in tobacco it has been suggested that CP12 has a role in redox-mediated regulation of carbon partitioning from the chloroplast [36]. In cyanobacteria it was shown that CP12 regulation occurs via the NAD(H)/NADP(H) ratio under light/dark conditions [37]. This is noteworthy, since the NAD Kinase, the sole source of NADP production, in higher plants is a calmodulin-binding protein and thus supposedly regulated by calcium [38].

Together, calcium and redox regulation have been shown to influence other activities of Calvin-Benson-Bassham Cycle enzymes as well. When reduced, fructose 1,6-bisphosphatase (FBP) activity was shown to be identical to oxidized fructose FBP pre-incubated with calcium [39]. In a similar way, sedoheptulose l,7-biphosphatase (SBP) becomes activated when reduced by thioredoxin upon dark-light transitions [40]. Furthermore, catalytic efficiency of both enzymes is inhibited by high calcium concentrations [41,42,43]. Upon light-dark transition, a transient increase in stromal free calcium concentration has been shown [44] and calcium was suggested to be actively transported into the lumen during the day to prevent calcium-dependent inhibition of CO_2_-fixation [45]. Thus calcium binding to CP12 could be part of a differential regulatory circuit of chloroplast carbon metabolism.

## References

[1] Berridge M.J., Lipp P., Bootman M.D. (2000). The versatility and universality of calcium signalling. Nat. Rev. Mol. Cell Biol..

[2] Clapham D.E. (2007). Calcium signaling. Cell.

[3] Rocha A.G., Vothknecht U.C. (2012). The role of calcium in chloroplasts—An intriguing and unresolved puzzle. Protoplasma.

[4] Heredia P., de Las Rivas J. (2003). Calcium-dependent conformational change and thermal stability of the isolated PsbO protein detected by FTIR Spectroscopy. Biochemistry.

[5] Kruk J., Burda K., Jemiola-Rzeminska M., Strzalka K. (2003). The 33 kDa protein of photosystem II is a low-affinity calcium- and lanthanide-binding protein. Biochemistry.

[6] Shutova T., Nikitina J., Deikus G., Andersson B., Klimov V., Samuelsson G. (2005). Structural dynamics of the manganese-stabilizing protein-effect of pH, calcium, and manganese. Biochemistry.

[7] Surek B., Kreimer G., Melkonian M., Latzko E. (1987). Spinach ferredoxin is a calcium-binding protein. Planta.

[8] Han S.C., Tang R.H., Anderson L.K., Woerner T.E., Pei Z.M. (2003). A cell surface receptor mediates extracellular Ca^2+^ sensing in guard cells. Nature.

[9] Nomura H., Komori T., Kobori M., Nakahira Y., Shiina T. (2008). Evidence for chloroplast control of external Ca^2+^-induced cytosolic Ca^2+^ transients and stomatal closure. Plant J..

[10] Tang R.H., Han S.C., Zheng H.L., Cook C.W., Choi C.S., Woerner T.E., Jackson R.B., Pei Z.M. (2007). Coupling diurnal cytosolic Ca^2+^ oscillations to the CAS-IP3 pathway in Arabidopsis. Science.

[11] Weinl S., Held K., Schlucking K., Steinhorst L., Kuhlgert S., Hippler M., Kudla J. (2008). A plastid protein crucial for Ca^2+^-regulated stomatal responses. New Phytol..

[12] Vainonen J.P., Sakuragi Y., Stael S., Tikkanen M., Allahverdiyeva Y., Paakkarinen V.,  Aro E., Suorsa M., Scheller H.V., Vener A.V. (2008). Light regulation of CaS, a novel phosphoprotein in the thylakoid membrane of Arabidopsis thaliana. FEBS J..

[13] Stael S., Rocha A.G., Wimberger T., Anrather D., Vothknecht U.C., Teige M. (2012). Cross-talk between calcium signalling and protein phosphorylation at the thylakoid. J. Exp. Bot..

[14] Nakayama S., Kretsinger R.H. (1994). Evolution of the EF-hand family of proteins. Ann. Rev. Biophys. Biomol. Struct..

[15] Reddy V.S., Ali G.S., Reddy A.S.N. (2002). Genes encoding calmodulin-binding proteins in the arabidopsis genome. J. Biol. Chem..

[16] Yang T., Poovaiah B.W. (2000). Arabidopsis chloroplast chaperonin 10 is a calmodulin-binding protein. Biochem. Biophys. Res. Commun..

[17] Buaboocha T., Liao B., Zielinski R.E. (2001). Isolation of cDNA and genomic DNA clones encoding a calmodulin-binding protein related to a family of ATPases involved in cell division and vesicle fusion. Planta.

[18] Bussemer J., Chigri F., Vothknecht U.C. (2009). Arabidopsis ATPase family gene 1-like protein 1 is a calmodulin-binding AAA^+^-ATPase with a dual localization in chloroplasts and mitochondria. FEBS J..

[19] Bayer R.G., Stael S., Csaszar E., Teige M. (2011). Mining the soluble chloroplast proteome by affinity chromatography. Proteomics.

[20] Ferro M., Salvi D., Brugiere S., Miras S., Kowalski S., Louwagie M., Garin J., Joyard J., Rolland N. (2003). Proteomics of the chloroplast envelope membranes from Arabidopsis thaliana. Mol. Cell. Proteomics.

[21] Stael S., Rocha A.G., Robinson A.J., Kmiecik P., Vothknecht U.C., Teige M. (2011). Arabidopsis calcium-binding mitochondrial carrier proteins as potential facilitators of mitochondrial ATP-import and plastid SAM-import. FEBS Lett..

[22] Tozawa Y., Nozawa A., Kanno T., Narisawa T., Masuda S., Kasai K., Nanamiya H. (2007). Calcium-activated (p)ppGpp synthetase in chloroplasts of land plants. J. Biol. Chem..

[23] Wilkins M.R., Gasteiger E., Bairoch A., Sanchez J.C., Williams K.L., Appel R.D., Hochstrasser D.F. (1999). Protein identification and analysis tools in the ExPASy server. Methods Mol. Biol..

[24] Emanuelsson O., Nielsen H., von Heijne G. (1999). ChloroP, a neural network-based method for predicting chloroplast transit peptides and their cleavage sites. Protein Sci..

[25] Seigneurin-Berny D., Salvi D., Dorne A.J., Joyard J., Rolland N. (2008). Percoll-purified and photosynthetically active chloroplasts from Arabidopsis thaliana leaves. Plant Physiol. Biochem..

[26] Maruyama K., Mikawa T., Ebashi S. (1984). Detection of calcium-binding proteins by ^45^Ca autoradiography on nitrocellulose membrane after sodium dodecyl-sulfate gel-electrophoresis. J. Biochem..

[27] Wedel N., Soll J., Paap B.K. (1997). CP12 provides a new mode of light regulation of Calvin cycle activity in higher plants. Proc. Natl. Acad. Sci. USA.

[28] Wedel N., Soll J. (1998). Evolutionary conserved light regulation of Calvin cycle activity by NADPH-mediated reversible phosphoribulokinase/CP12/glyceraldehyde-3-phosphate dehydrogenase complex dissociation. Proc. Natl. Acad. Sci. USA.

[29] Lebreton S., Graciet E., Gontero B. (2003). Modulation, via protein-protein interactions, of glyceraldehyde-3-phosphate dehydrogenase activity through redox phosphoribulokinase regulation. J. Biol. Chem..

[30] Nicholson S., Easterby J.S., Powls R. (1987). Properties of a multimeric protein complex from chloroplasts possessing potential activities of NADPH-dependent glyceraldehyde-3-phosphate dehydrogenase and phosphoribulokinase. Eur. J. Biochem..

[31] Obrien M.J., Easterby J.S., Powls R. (1976). Algal glyceraldehyde-3-phosphate dehydrogenases conversion of NADH-linked enzyme of scenedesmus-obliquus into a form which preferentially uses NADPH as coenzyme. Biochim. Biophys. Acta.

[32] Boggetto N., Gontero B., Maberly S.C. (2007). Regulation of phosphoribulokinase and glyceraldehyde 3-phosphate dehydrogenase in a freshwater diatom, Asterionella formosa. J. Phycol..

[33] Erales J., Gontero B., Maberly S.C. (2008). Specificity and function of glyceraldehyde-3-phosphate dehydrogenase in a freshwater diatom, Asterionella formosa (Bacillariophyceae). J. Phycol..

[34] Marri L., Zaffagnini M., Collin V., Issakidis-Bourguet E., Lemaire S.D., Pupillo P., Sparla F., Miginiac-Maslow M., Trost P. (2009). Prompt and easy activation by specific thioredoxins of Calvin cycle enzymes of Arabidopsis thaliana associated in the GAPDH/CP12/PRK supramolecular complex. Mol. Plant.

[35] Graciet E., Lebreton S., Camadro J.-M., Gontero B. (2003). Characterization of native and recombinant A4 glyceraldehyde 3-phosphate dehydrogenase. Eur. J. Biochem..

[36] Howard T.P., Fryer M.J., Singh P., Metodiev M., Lytovchenko A., Obata T., Fernie A.R., Kruger N.J., Quick W.P., Lloyd J.C. (2011). Antisense suppression of the small chloroplast protein CP12 in tobacco alters carbon partitioning and severely restricts growth. Plant Physiol..

[37] Tamoi M., Miyazaki T., Fukamizo T., Shigeoka S. (2005). The Calvin cycle in cyanobacteria is regulated by CP12 via the NAD(H)/NADP(H) ratio under light/dark conditions. Plant J..

[38] Turner W.L., Waller J.C., Vanderbeld B., Snedden W.A. (2004). Cloning and characterization of two NAD kinases from Arabidopsis. Identification of a calmodulin binding isoform. Plant Physiol..

[39] Chardot T., Meunier J.-C. (1990). Fructose-1,6-bisphosphate and calcium activate oxidized spinach (Spinacia oleracea) chloroplast fructose-1,6-bisphosphatase. Plant Sci..

[40] Cadet F., Meunier J.C. (1988). Spinach (Spinacia oleracea) chloroplast sedoheptulose-1,7-bisphosphatase. activation and deactivation, and immunological relationship to fructose-1,6-bisphosphatase. Biochem. J..

[41] Charles S.A., Halliwell B. (1980). Action of calcium-ions on spinach (Spinacia-Oleracea) chloroplast fructose bisphosphatase and other enzymes of the Calvin cycle. Biochem. J..

[42] Portis A.R., Heldt H.W. (1976). Light-dependent changes of the Mg^2+^ concentration in the stroma in relation to the Mg^2+^ dependency of CO_2_ fixation in intact chloroplasts. Biochim. Biophys. Acta Bioenerg..

[43] Wolosiuk R.A., Hertig C.M., Nishizawa A.N., Buchanan B.B. (1982). Enzyme regulation in C_4_ photosynthesis. Role of Ca^2+^ in thioredoxin-linked activation of sedoheptulose bisphosphatase from corn leaves. FEBS Lett..

[44] Sai J., Johnson C.H. (2002). Dark-stimulated calcium ion fluxes in the chloroplast stroma and cytosol. Plant Cell.

[45] Ettinger W.F., Clear A.M., Fanning K.J., Peck M.L. (1999). Identification of a Ca^2+^/H^+^ antiport in the plant chloroplast thylakoid membrane. Plant Physiol..

